# Nanoantenna-based ultrafast thermoelectric long-wave infrared detectors

**DOI:** 10.1038/s41598-020-70062-6

**Published:** 2020-08-10

**Authors:** Gergo P. Szakmany, Gary H. Bernstein, Edward C. Kinzel, Alexei O. Orlov, Wolfgang Porod

**Affiliations:** 1 Department of Electrical Engineering, University of Notre Dame, Notre Dame, IN 46556 USA; 2 Department of Aerospace and Mechanical Engineering, University of Notre Dame, Notre Dame, IN 46556 USA

**Keywords:** Imaging and sensing, Nanosensors

## Abstract

We investigate the generation of electrical signals by suspended thermoelectrically coupled nanoantennas (TECNAs) above a quasi-spherical reflector cavity in response to rapidly changing long-wave infrared radiation. These sensors use a resonant nanoantenna to couple the IR energy to a nanoscale thermocouple. They are positioned over a cavity, etched into the Si substrate, that provides thermal isolation and is designed as an optical element to focus the IR radiation to the antenna. We study the frequency-dependent response of such TECNAs to amplitude-modulated 10.6 μm IR signals. We experimentally demonstrate response times on the order of 3 μs, and a signal bandwidth of about 300 kHz. The observed electrical response is in excellent correlation with finite element method simulations based on the thermal properties of nanostructures. Both experiments and simulations show a key trade-off between sensitivity and response time for such structures and provide solutions for specific target applications.

## Introduction

Conventional wisdom states that uncooled thermal IR sensors, such as bolometers and thermocouples, must be slow and/or insensitive. In these sensors, both the absorbed power and thermal mass scale with the physical volume of the device. As a consequence, reductions in pixel size increase the cutoff frequency, but they also reduce the sensitivity to IR irradiation. This tradeoff limits the frequency response of traditional microbolometers to about 30–60 Hz^1–3^. Umbrella-type microbolometers^4^ having a double-layer structure attempt to overcome this limitation by using a space-filling metal/dielectric layer to maximize the absorbed IR energy^5^. Punctured holes in the space-filling umbrella layer reduce the thermal mass of these devices, and as a result their frequency response increases to a few 100 Hz^1^. Here, we demonstrate that uncooled thermal infrared detectors can be extremely fast while their sensitivity is relatively maintained.

In this paper, we study the frequency-dependent response of suspended thermoelectrically coupled nanoantennas (TECNAs) for long-wave infrared (LWIR) detection. TECNAs are radiation-field type LWIR detectors; the freely propagating electromagnetic waves are resonantly absorbed by a nanoantenna. Radiation-induced antenna currents lead to Joule heating of the hot junction of a nanothermocouple (NTC) at the center of the antenna^6–8^, causing a temperature difference, $\Delta T$, between it and the cold junction. This $\Delta T$ generates a thermoelectric voltage that is proportional to the intensity of the incident IR radiation. The physical size and shape of the antenna determine the spectral and polarization properties of the device. In this paper we present TECNAs that are sensitive to 28.3 THz (10.6 μm) IR signals.

The TECNAs reported here are suspended over a quasi-hemispherical cavity etched into the Si substrate; the nanoantenna and the hot junction are not in direct thermal contact with the substrate. The etched cavity beneath the antenna thermally isolates the antenna^9,10^ while also focusing reflected IR radiation onto the antenna. We have previously shown that these combined effects result in about a 100 times enhancement of the thermal response compared to TECNAs on a SiO_2_/Si substrate^11,12^, where the nanoantenna and the hot junction are in direct contact with the substrate. Simulations predict that the electromagnetic focusing effect accounts for a 7.5 × increase of the effective antenna aperture area when the reflector is defined in Si (despite the Fresnel reflectance of ~ 0.3 at normal incidence).

The NTC, which converts the thermal energy to electrical signals, can be constructed by either two dissimilar metals, as usual, or as we have introduced^13^^,^ from a single metal layer by utilizing the size dependence of electronic transport at the nanoscale^14–16^. In particular, the required non-zero relative Seebeck coefficient of the NTC is achieved by using two metallic nanowire segments having different cross sections. The two different metallic nanowire segments behave like two different metals that exhibit distinct absolute Seebeck coefficients, creating a thermocouple junction at the size discontinuity^13,15–17^.

Practical applications for IR sensing might require multiple TECNAs in an array. To this end, we have also demonstrated that suspended TECNAs can be arranged in a thermopile by connecting them in series^18^. As a result, the SNR of the nanoantenna thermopile increases by $\sqrt N$, where *N* is the number of TECNAs in the thermopile. This also assumes spatially uniform illumination of the *N* TECNAs in the thermopile.

In this paper, we study the frequency-dependent thermoelectric response of TECNAs to amplitude-modulated LWIR signal excitation. Amplitude modulation was achieved by an acousto-optic modulator (AOM). For TECNAs, the absorption cross-section corresponds to the effective aperture area of the resonant antenna (the effective absorptivity of the resonant antenna can exceed unity). For a thin metal dipole antenna, the thermal mass is about 25 fJ/K as discussed below. In this case, the unusually large ratio of the effective absorption area to the thermal mass corresponds to a dramatic increase in the traditional tradeoff between responsivity and cutoff frequency and allows TECNAs to approach the performance of cooled devices, which contradicts the conventional wisdom that thermal IR sensors must be slow and/or insensitive. In this paper we demonstrate that the very low thermal mass leads to a very short response time—up to 1000 × faster than bolometers. We show ultrafast thermoelectric detection of amplitude modulated LWIR waves with a − 3 dB level at around 300 kHz.

## Design and fabrication

### Antenna design

The antenna is the receiving element that converts the optical energy through Joule heating by the radiation-induced antenna currents of the nanoantenna material. Based on COMSOL simulations and the optical properties of metals at infrared frequencies, Pd was selected as the material for both the antenna and the NTC. Its thermal conductivity is lower than that of Au and Ni, so more heat is produced while minimizing the thermal diffusion through the lead lines^19,20^. Maximum heating occurs when the antenna is at resonance, i.e., its length matches the effective wavelength of the incident radiation. For IR radiation at 10.6 μm, the resonant antenna length of a suspended IR antenna is 3.5 μm, as determined by COMSOL simulations. This provides an effective antenna aperture area of *P*_*abs*_/*E*_0_ = 5.13 μm^2^ which is slightly less than that for an ideal isotropic antenna (λ^2^/4π). When the cavity is included in the simulation the effective aperture rises to 38.5 μm^2^. This corresponds to an aperture efficiency of 22.6%. This can be improved by increasing the reflectivity of the cavity.

**Figure 1:**
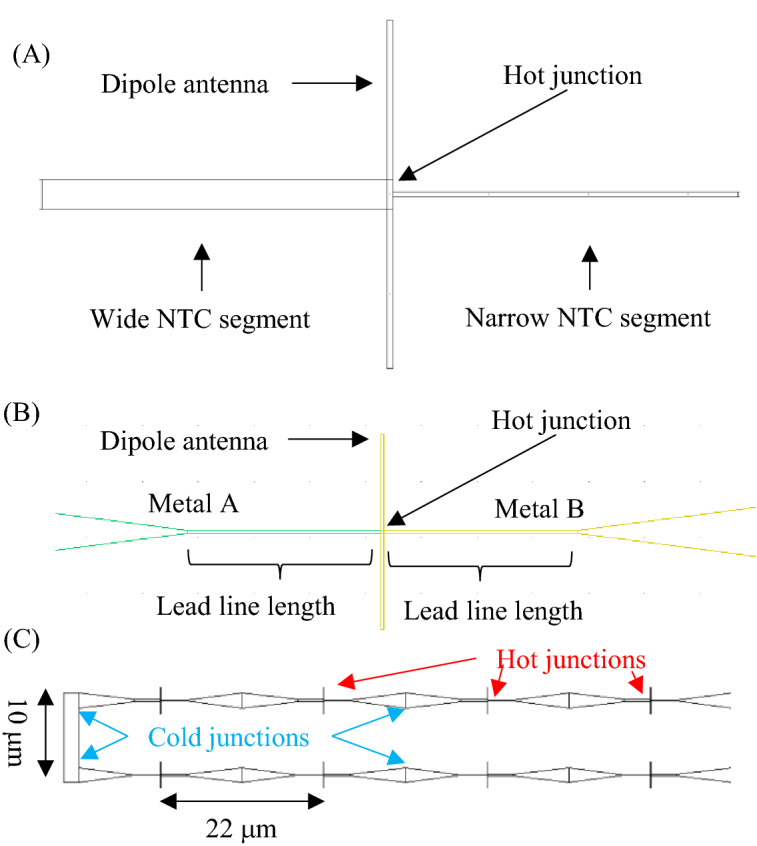
Schematics of antenna elements and thermopiles. (**A**) An individual single-metal TECNA, (**B**) an individual bi-metal TECNA, and (**C**) segment of a thermopile constructed from single-metal TECNAs.

At IR wavelengths, the conductivity of metals is significantly lower than at RF/microwave frequencies. As a result, significantly more heat is produced by the antenna. The thermal energy is converted to DC electrical signals by the NTC. For a dipole antenna, the current maximum, and thus the maximum heating, occurs at its center. Therefore, the hot junction of the NTC must be placed at the center of the antenna. Based on our previous work^13^^,^ single-metal NTCs were constructed from a single layer of Pd using narrow (50 nm) and wide (300 nm) wire segments that result in a relative Seebeck coefficient, *ΔS* = 1.2 μV/K^13^^,^ as shown in Fig. 1a. The length of the narrow and wide wire segments was 3.5 μm.

Bi-metal TECNAs were constructed from 50-nm-wide Pd and Ni wire segments (*ΔS* = 8.9 μV/K)^17^ as shown in Fig. 1b. Bi-metal TECNAs with two different lead line lengths, 1 μm and 3.5 μm were built.

### Nanoantenna thermopile design

In order to construct a nanoantenna thermopile, nominally identical single-metal TECNAs were connected in series at their cold junctions; the cold junctions are situated outside of the cavity and form a good thermal contact with the substrate. The horizontal and vertical spacings of the antennas are 22 μm and 10 μm, respectively, as shown in Fig. 1c. Due to limited space imposed by the bonding pads on our chip, nanoantenna thermopiles with 52 TECNAs were selected. This gives a total sensor area of 200 × 300 μm^2^.

### Fabrication

We use a high-resistivity Si (> 20,000 Ω cm) wafer for the substrate of the TECNAs and thermopiles. This ensures electrical isolation between the devices, and avoids the need of an insulating oxide layer that created mechanical stress and subsequent failure of the TECNAs^21^. In addition, the oxide absorbs the CO_2_ laser irradiation used during the measurements, and as a result increases the thermal stress in the devices. The bonding pads and the lead lines for the electrical measurements were patterned by optical lithography and formed from a 200-nm-thick Au layer using 10 nm Ti as an adhesion layer.

Fabrication of single-metal TECNAs and nanoantenna thermopiles was accomplished using a single step of electron beam lithography (EBL) and metal deposition. The fabrication starts with the cleaning of the substrate for 30 s in hydrogen fluoride (HF) to remove any native oxide from the Si surface. Then, the antenna and NTCs were patterned by a Raith EBPG 5200 electron beam lithography system into a polydimethylglutarimide polymer (PMGI) and polymethyl methacrylate (PMMA) double-layer resist. First, the PMMA layer was developed in a mixture of MIBK, IPA, and MEK^22^. Then the PMGI was removed under the developed areas using AZ 917 developer for 10 s. This also creates a desired undercut in the resist profile for the lift-off process. The devices were formed from a 45-nm-thick Pd layer using 5 nm Ti as an adhesion layer. Lift-off was performed in 1-methyl-2-pyrrolidinone (NMP).

The fabrication of the bi-metal TECNAs is similar. After the antennas and one arm of the NTCs were fabricated as discussed above, the second arm was patterned by EBL and formed from a 50-nm-thick Ni layer.

**Figure 2:**
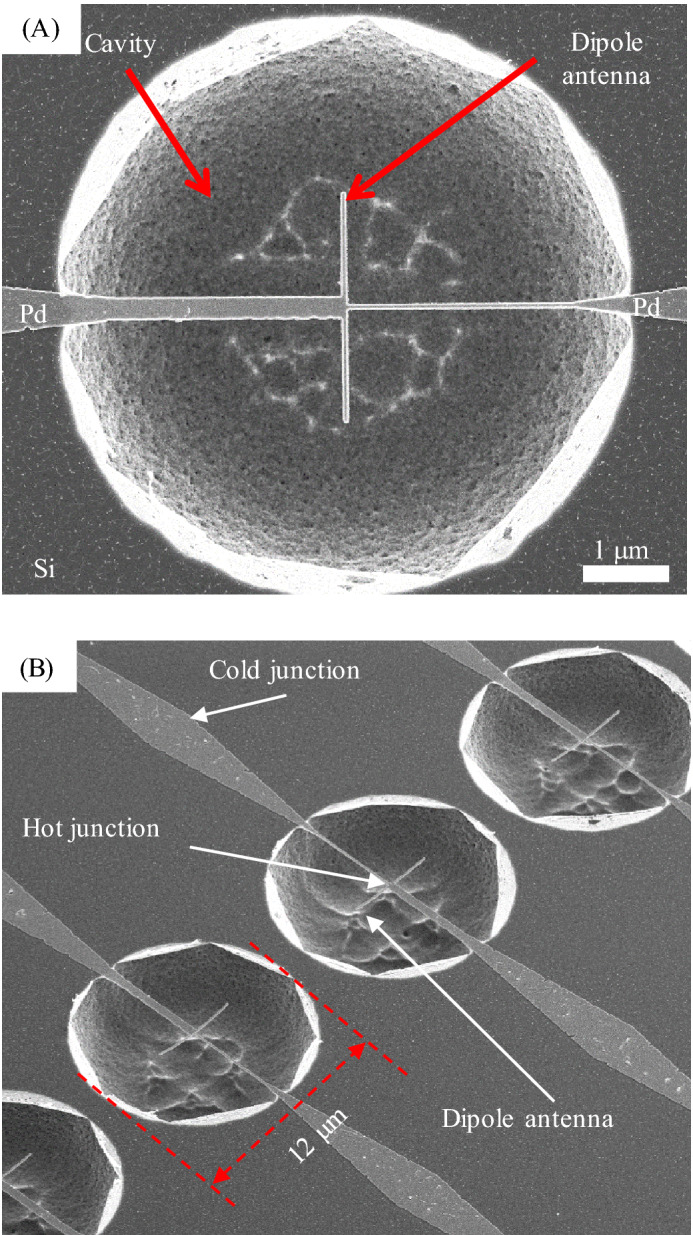
SEM images of a thermopile. (**A**) Top view of a section of a suspended single-metal TECNA above a cavity and (**B**) tilted view of a few devices in a nanoantenna thermopile.

After the TECNAs were fabricated, 6-μm-diameter circles centering around the antenna were exposed into a PMMA resist layer. After development, cavities were formed under the TECNAs by using a Memsstar BT001 XeF_2_ vapor etch system at 3.5 torr for 15 s. The isotropic nature of the XeF_2_ etch creates quasi-hemispherical cavities, as shown in Fig. 2. The radius of the cavity aperture is 12 μm, and the cavity under the antenna is 4.9 μm deep. More details of the suspended TECNA fabrication process can be found in^21^.

## Results

For IR testing, the fabricated devices were wire bonded to a chip carrier, and the devices were positioned using linear stages to be at the center of a normally incident CO_2_ laser beam. The devices were illuminated by a CO_2_ laser operating at 10.6 μm (28.3 THz) with an intensity of 1.42 W/cm^2^. The laser beam was amplitude modulated by an AOM up to 6 MHz. The modulation efficiency loss within this range was less than 10% for a beam diameter at the input of the AOM of about 250 μm. For reference, the AOM modulated signal was also measured by an uncooled MCT detector, Boston PVM-10.6-1 × 1, using lock-in techniques. This measurement revealed that the modulation efficiency of the first diffracted beam was constant within 8% at least up to 5 MHz. This allowed us to assume a constant modulation strength of the incident IR irradiation for the frequency range over which our devices were investigated. The TECNA response, an open-circuit voltage, *V*_*OC*_, was first boosted by a broad-band differential pre-amplifier with a − 3 dB bandwidth of 12 MHz and then detected with a lock-in amplifier. The preamplifier was directly soldered to the IC socket that holds the chip carrier with the wire-bonded sample to minimize a parasitic time constant formed by resistance of the thermopile (~ 30 kΩ) and amplifier input capacitance (~ 3.5 pF).

For a dipole antenna, maximum response is achieved when the polarization of the incident IR wave is parallel to the long antenna axis^23^. This condition was set by a half-wave plate mounted in a stepper-motor-driven stage.

**Figure 3:**
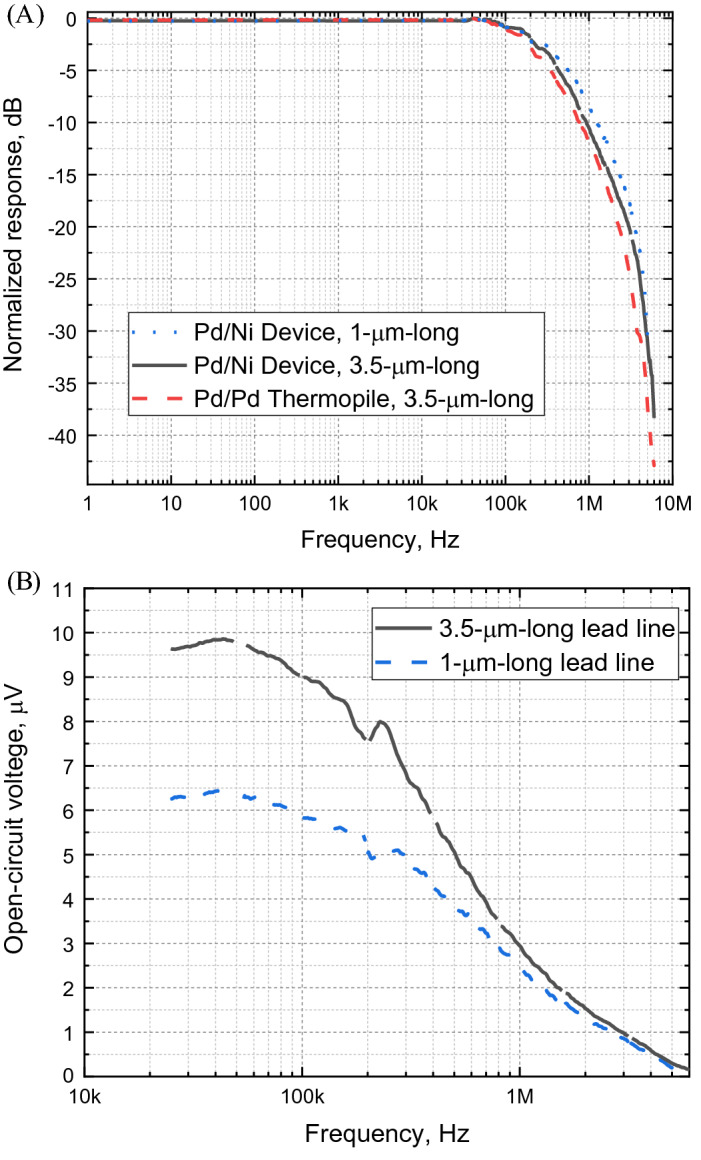
Frequency-dependent responses. (**A**) Normalized responses of bi-metal TECNAs with two different lead-line lengths and a nanoantenna thermopile comprising 52 single-metal TECNAs in a series connection. (**B**) The measured V_OC_ response as a function of laser beam modulation frequency. TECNAs with shorter lead lines offer higher bandwidth at the expense of poorer sensitivity.

Figure 3a shows the experimentally obtained frequency-dependent response of the suspended bi-metal TECNAs with two different lead-line lengths along with the suspended single-metal nanoantenna thermopile between 1 Hz and 6 MHz. We observe a steady, frequency-independent, signal in the AOM frequency range of 1 Hz to 70 kHz. At higher modulation frequencies the magnitude of the generated thermoelectric signals starts to decline, reaching a -3 dB level at 200 kHz for the thermopile, and at 300 kHz and at 370 kHz for the bi-metal TECNAs with 3.5 μm and 1 μm lead line lengths, respectively. Special care is taken to rule out possible signal reduction due to the low-pass filter formed by the series connection of the resistive TECNA element and input capacitance of the amplifier. It is determined that the electrical − 3 dB cutoff frequency is above 1.5 MHz.

The data of Fig. 3a strongly suggest that the electrical cutoff frequency of the amplifier is not the limit. Indeed, the electrical cutoff frequency for the single bi-metal devices would have been 30 times higher (~ 6 MHz) than that of the thermopile, which has a total series resistance that is 30 times larger than that of a single device. By contrast, the highest cutoff frequency experimentally observed for the 1 µm lead line bi-metal device, 370 kHz is less than two times higher than that of the thermopile (200 kHz). As a conclusion, thermal rather than electrical limitations set the maximal speed of the TECNA to the change in IR intensity.

Figure 3b illustrates the trade-off between sensitivity and bandwidth of TECNAs. The cutoff frequency of devices with 1-μm-long lead lines is higher than that of devices with 3.5-μm-long lead lines because the thermal resistance of the shorter wire segments is smaller (while their electrical resistance ratio is about 1.3). As a result, the cooling of the hot junction is more effective at the OFF state of the laser beam modulation, resulting in a faster electrical response. However, at the ON state of the modulation, the heat produced by the antenna is also removed more efficiently, resulting in a smaller temperature increase and a smaller generated *V*_*OC*_.

## Discussion

**Figure 4:**
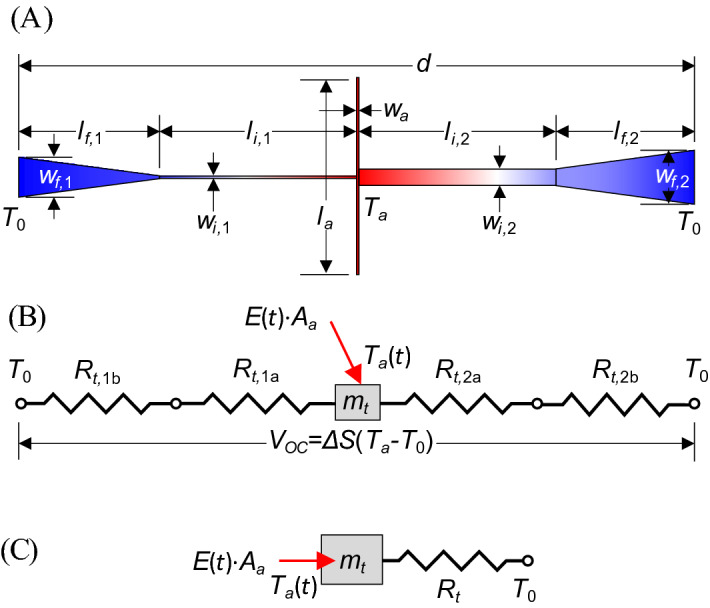
Simplified thermal model of TECNAs. (**A**) Antenna geometry, (**B**) thermal resistance model, and (**C**) final equivalent thermal circuit.

Here, we present a thermal analysis of these devices. Figure 4 shows a simplified thermal model of the TECNA. Adapting the standard thermal analysis used for microbolometers^24^ assumes that (1) there are negligible thermal losses to convection or radiation exchange with the environment (other than the incident laser beam), (2) the temperature of the antenna/junction is nearly uniform (lumped capacitance formulation), (3) the conduction along the feed lines is one dimensional and (4) the thermal energy stored in the feed structure is negligible. For a harmonic excitation with angular frequency, *ω*, the temperature difference between the antenna, *T*_*a*_, and the substrate, *T*_0_, *T*_*a*_ − *T*_0_ = Δ*T*, is given by

1a $$\Delta T = {{R_{t} A_{a} E} \mathord{\left/ {\vphantom {{R_{t} A_{a} E} {\sqrt {1 + \omega^{2} \tau_{t}^{2} } }}} \right. \kern-\nulldelimiterspace} {\sqrt {1 + \omega^{2} \tau_{t}^{2} } }}$$

1b $${\text{so}}\;{\text{that}}\;\Delta T_{0} = \Delta T_{\omega \to 0} = R_{t} A_{a} E,$$

where *A*_*a*_ is the aperture area of the antenna, *E* is the irradiance (incident power/area), *R*_*t*_ is the thermal resistance, and *τ*_*t*_ is the thermal time constant or thermal response time. The responsivity is given by the ratio of the open circuit voltage, *V*_*OC*_ = Δ*S*Δ*T*, to the irradiance

2a $$\Re_{V} = \frac{{V_{OC} }}{E} = N\frac{{R_{t} A_{a} \Delta S}}{{\sqrt {1 + \omega^{2} \tau_{t}^{2} } }}$$

2b $${\text{so}}\;{\text{that}}\;\Re_{V,0} = \Re_{V,\omega \to 0} = NR_{t} A_{a} \Delta S,$$

where Δ*S* is the difference of the Seebeck coefficients of the materials forming the junction at the antenna and *N* is the number of antennas in series constituting the thermopile array. This scales linearly with the thermal resistance.

The thermal resistance is defined as the temperature difference per thermal energy transported through the material per time and is typically expressed in K/W. The thermal resistance for a rectangular feed line is given by

3a $$R_{t,na} = \frac{{l_{i,n} }}{{k_{n} \cdot w_{i,n} h}},$$

where *k*_*n*_ is the thermal conductivity, *w*_*i,n*_ is the width, and *l*_*i,n*_ is the length (shown in Fig. 4), and the numerical subscripts, *n*, are used to indicate the respective legs. *h* is the thickness of the leads, which is assumed to be consistent throughout the device. For the tapered section, the width changes over the length and has resistance

3b $$R_{t,nb} = \frac{{l_{f,n} }}{{k_{n} \cdot h\left( {w_{f,n} - w_{i,n} } \right)}}\ln \left( {\frac{{w_{f,n} }}{{w_{i,n} }}} \right),$$

where *l*_*f,n*_ is the length (taken as *l*_*f,n*_ = *d*/2 − *w*_*a*_/2 − *l*_*i,n*_), and *w*_*f,n*_ is the width at of the lead line where it makes contact with the substrate at the edge of the cavity. The equivalent thermal resistance of one lead is found by placing the resistances of the rectangular and tapered section in series. The two leads are in parallel and combine to provide an equivalent thermal resistance of

3c $$R_{t} = \frac{{\left( {R_{t,1a} + R_{t,1b} } \right)\left( {R_{t,2a} + R_{t,2b} } \right)}}{{\left( {R_{t,1a} + R_{t,1b} } \right) + \left( {R_{t,2a} + R_{t,2b} } \right)}},$$

It should be noted that the DC electrical resistance of the structure can be calculated by substituting the electrical conductivity for the thermal conductivity and taking the series combination of the four terms.

The thermal mass is defined as the thermal energy required to raise the temperature of the antenna and is equivalent to the thermal capacitance of the system

4a $$m_{t} = c_{p} \rho V,$$

where *c*_*p*_ is the specific heat, *ρ* is the density, and *V* is the volume (all of the antenna). The thermal mass of the 3.5 × 0.5 × 0.5 µm^3^ Pd antennas in this paper is *m*_*t*_ = 2.56 × 10^–14^ J/K. The time constant for the 1st order thermal system is given by

4b $$\tau_{t} = m_{t} R_{t} = c_{p} \rho VR_{t} ,$$

which, from Eq. (2a), corresponds to a − 3 dB cutoff frequency of

4c $$f_{c} \approx \frac{1}{{2\pi \tau_{t} }}.$$

**Figure 5:**
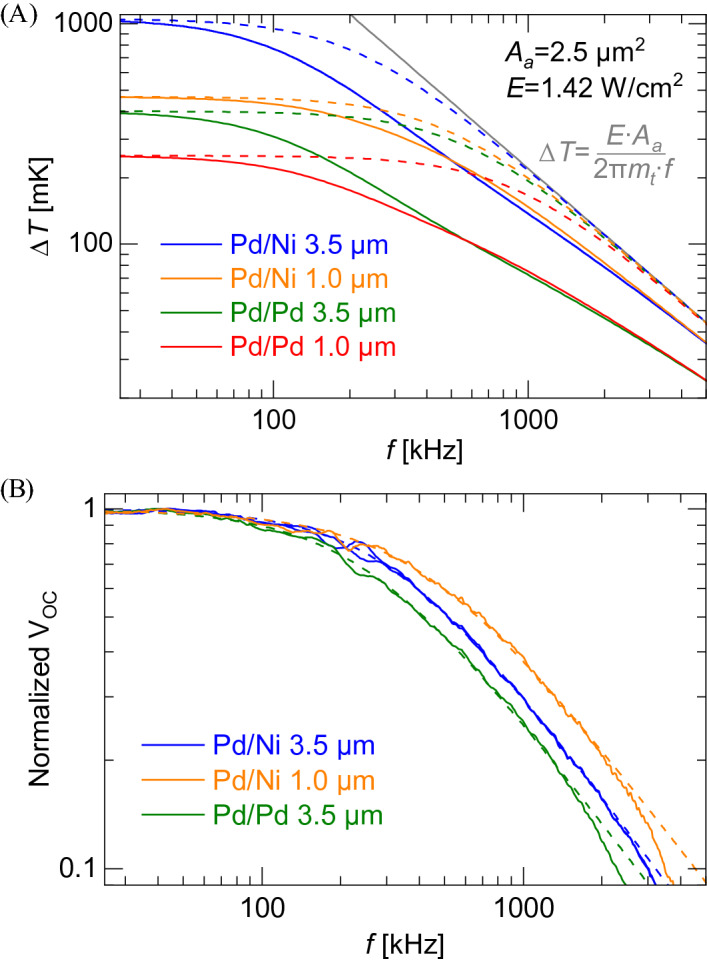
Thermal analysis of TECNAs. (**A**) Simulated and modeled dynamic response and (**B**) experimental data fitted with analytical model.

Figure 5a and Table 1 give the modeled response for the four different feed structures. Figure 5a also shows the results from finite element method (FEM) simulations (ANSYS Mechanical) of the harmonic response. The FEM results match the lumped capacitance results at low frequencies (Eq. (1b)). However, at higher frequencies, the lumped capacitance solutions converge to Δ*T* = *A*_*a*_*E*/(2π*m*_*t*_*f*) from Eqs. (1a) and (4b), irrespective of the feed structure. The FEM results capture the thermal energy stored in the lead lines, which significantly contributes to the thermal mass of the system. Unlike microbolometers, this effect is significant because of the extremely low thermal mass of the antenna/junction (Eq. (4a)). Some insight is gained by considering the thermal diffusion length into the lead lines, which is defined as

5 $$\delta = \sqrt {{{2\alpha } \mathord{\left/ {\vphantom {{2\alpha } \omega }} \right. \kern-\nulldelimiterspace} \omega }} ,$$

**Table 1:** Cutoff frequency (kHz) for lumped capacitance and FEM simulations.

Design	Pd/Pd	Pd/Pd	Pd/Ni	Pd/Ni
*l*_*i*_ [µm]	1.0	3.5	1.0	3.5
*R*_*t*_ [K/W]	7.10 × 10^6^	1.13 × 10^7^	1.31 × 10^7^	2.97 × 10^7^
*f*_*c,LC*_ [kHz]	958	550	514	209
*f*_*c,FEM*_ [kHz]	213	102	291	78.2
*f*_*c,exp*_ [kHz]	–	208	367	287
*V*_*OC,LC*_ [µV]	0.302	0.481	4.15	9.37
*V*_*OC,LC*_ [µV]	–	0.481	4.52	7.00

where the thermal diffusivity is *α* = *k*/*ρ*·*c*. For example, *δ* = 2.18 µm for a Pd antenna at 952 kHz and 5.06 µm at 102 kHz. The extended thermal mass can be included in Eq. (2a) as

6 $$\Re_{V} = \frac{{NR_{t} A_{a} \Delta S}}{{\sqrt {1 + \omega^{2} R_{t}^{2} \left( {m_{t,0} + c_{1} \sqrt {{{2\alpha } \mathord{\left/ {\vphantom {{2\alpha } \omega }} \right. \kern-\nulldelimiterspace} \omega }} } \right)} }} = \frac{{c_{2} }}{{\sqrt {1 + c_{3} f^{2} + c_{4} f^{1.5} } }},$$

where *c*_1_ ~ *hwρc*_*p*_ and scales the thermal diffusion length by the cross-sectional area of the leads along with the density and specific heat of the metal. The functional form of the equation allows *c*_2_, *c*_3_, and *c*_4_ to be fit to experimental data. Figure 5b shows that the model in Eq. (6) (dashed lines) fits very well to the normalized open circuit data for the antennas. A comparison between measured and modeled cutoff data is reported in Table 1 along with the low-frequency open-circuit voltage (based on fitting the aperture area to be *A*_*a*_ = 2.5 µm^2^). These results show that good first order estimates of the performance can be achieved from the simplified 1^st^ order model, but that thermal diffusion produces a cutoff frequency 50% smaller than predicted from models.

The relationship between the low-frequency responsivity and the cutoff frequency is given by Eqs. (2b) and (4b) after solving for the thermal resistance,

7 $$\Re_{V,0} = \frac{N}{2\pi }\Delta S\left( {\frac{{A_{a} }}{{m_{t} }}} \right)\frac{1}{{f_{c} }}.$$

This tradeoff does not account for thermal diffusion into the lead lines, but shows that the ratio of the effective aperture area of the antenna to its thermal mass is a figure of merit for the design. The impact of the thermal diffusion into the feed lines can be minimized by keeping the width of the feed lines adjacent to the antenna small. Beyond this, the proportionality of the electrical resistance to the thermal resistance recommends keeping the overall thermal resistance low to minimize thermal noise^24^.

## Summary and conclusion

We have shown that suspended thermoelectrically coupled nanoantennas and nanoantenna thermopiles are capable of fast response to rapidly changing IR signals with a response time < 3 μs despite the conventional wisdom that thermal based IR sensors (bolometer and thermocouple) are slow and bulky. Even shorter response time can be achieved at the expense of sensitivity. The TECNAs are suspended in air above a quasi-spherical cavity etched into the Si substrate. The fast operating speed is achieved by resonant absorption of the IR radiation using a dipole antenna. Therefore, the size of the thermoelectric transducer must be very small to ensure small thermal mass.

Thermal analysis shows good agreement with the experimental results and reveals that the ratio of thermal diffusion into the NTC lead lines to the thermal mass of the system which determines the frequency response of the devices.

We have previously demonstrated that NTCs fabricated directly on a substrate are able to detect thermal oscillations up to 20 MHz^25^. Here we show that a lower cutoff frequency of suspended TECNAs correlates well with much slower heat removal due to thermal isolation from the substrate. However, the sensitivity of the suspended design is greatly enhanced (by a factor of ~ 100) compared to TECNAs that are in direct thermal contact with a Si/SiO_2_ substrate.

The measured specific detectivity of these devices is on the order of $5 \times 10^{7} \, \frac{{{\text{cm}}\,\sqrt {{\text{Hz}}} }}{{\text{W}}}$, which is about half an order of magnitude less sensitive than commercial bolometer-based mid-IR sensors. The sensitivity of our devices can be significantly increased by improving the reflectance of the cavity and using high *ZT* materials for the thermocouples. However, these processes require more-complex fabrication, and we leave it for future work.

The sensitivity and the fast operating speed make these devices competitive with existing thermal IR detectors, having unique features that include polarization sensitivity and wavelength selectivity.

## Data Availability

The datasets generated during and/or analyzed during the current study are available from the corresponding author on reasonable request.
